# Antibacterial and anti-trichomunas characteristics of local landraces of *Lawsonia inermis* L.

**DOI:** 10.1186/s12906-022-03676-0

**Published:** 2022-07-30

**Authors:** M. Fatahi Bafghi, S. Salary, F. Mirzaei, H. Mahmoodian, H. Meftahizade, R. Zareshahi

**Affiliations:** 1 Department of Microbiology, School of Medicine, Shahid Sadoughi University of Medical Sciences, Yazd, Iran; 2 Herbal Medicines Research Center, Student Research Committee, School of Pharmacy, Shahid Sadoughi University of Medical Science Health Services, Yazd, Iran; 3 Department of Parasitology and Mycology, School of Medicine, Shahid Sadoughi University of Medical Sciences, Yazd, Iran; 4 Management Department, Meybod University, Meybod, Iran; 5 Department of Horticultural Science, Faculty of Agriculture & Natural Resources, Ardakan University, P.O. Box 184, Ardakan, Iran; 6 Department of Pharmacognosy, School of Pharmacy, Shahid Sadoughi University of Medical Sciences, Yazd, 8951737915 Iran; 7 Herbal Medicines Research Center, School of Pharmacy, Shahid Sadoughi University of Medical Sciences, Yazd, Iran

**Keywords:** *Lawsonia inermis*, *Streptococcus agalactiae*, *Pseudomonas aeruginosathats*, Antibiotic resistance, *Trichomonas vaginalis*

## Abstract

**Background:**

Henna (*Lawsonia inermis*) with anti-bacterial properties has been widely used in traditional medicine especially Persian medicine. Henna oil is suggested for diseases of infectious origin, such as cervical ulcers. Group B *Streptococcus agalactiae*, *Pseudomonas aeruginosa* and, *Trichomonas vaginalis* are involved in the infection of women especially cervicitis. Henna grows in dry and tropical regions. The main important landraces of henna landraces are cultivated in Kerman, Sistan and Baluchestan, Hormozgan, and Bushehr provinces in Iran.

Proper use of antimicrobial agents, use of new antimicrobial strategies, and alternative methods, such as herbal methods may help reduce drug resistance in the future. This study’s objective was to investigate the anti-*Trichomonas vaginalis* activity of three different henna landraces and antimicrobial effects against group B *Streptococcus agalactiae* and, *Pseudomonas aeruginosa*.

**Methods:**

Total phenol content was measured by Folin ciocaltu method. Antibacterial effect of landraces of Henna against *P. aeruginosa* and *S. agalactiae* were assayed by well diffusion method and minimal inhibitory concentration assessments were done using the broth micro-dilution technique. Anti-Trichomonas effect of Henna landraces were assayed by Hemocytometery method.

**Results:**

Total phenol content of Shahdad, Rudbar-e-Jonub, and Qaleh Ganj was 206.51, 201.96, and 254.85 μg/ml, respectively. Shahdad, Rudbar-e-Jonub, and Qaleh Ganj had MIC against GBS at 15, 15 and, 4 μg/ml. The growth inhibition diameter of the most effective henna (Shahdad landrace) at a concentration of 20 μg/ml on *P. aeruginosa* was 2.46 ± 0.15 cm and in the MIC method at a concentration of 5 μg/ml of Shahdad landrace, *P. aeruginosa* did not grow. IC50 of shahdad Henna after 24 h, 48 h, and 72 h was 7.54, 4.83 and 20.54 μg/ml, respectively. IC50 of Rudbar-e-Jonub extract was 5.76, 3.79 and 5.77 μg/ml in different days. IC50 of Qaleh Ganj extract was 6.09, 4.08 and 5.74 μg/ml in different days.

**Conclusions:**

The amount of total phenol in Qaleh Ganj was higher than the other varieties. In the well diffusion method, Qaleh Ganj was more effective against group B *Streptococcus* (Gram-positive bacterium) than the other two landraces, and Shahdad landrace was more effective against *P. aeruginosa* (Gram-negative bacterium) than other. In the MIC method, the same result was obtained as in the well diffusion method, but at a lower concentration.

## Background

Classically, herbs have been applied because of their antibacterial effects, which is because of bioactive principles [1]. Medicinal plants, like *Lawsonia inermis* have been employed as antimicrobial drugs to prevent the growth of multi-drug resistant bacteria [2]. The antimicrobial resistance-associated infectious diseases, such as hospital-acquired Gram-negative bacterial infection, and the resulting mortality and morbidity, have been increasing at an alarming rate. Many antibiotics have become ineffective in treating and controlling multidrug-resistant bacterial pathogens worldwide [3].

*Lawsonia inermis* L. (Lythraceae), known as henna, is found in tropical and subtropical areas and has long been used worldwide [4]. Iran is one of the habitats of this plant. The main important landraces of henna are cultivated in Kerman, Sistan and Baluchestan, Hormozgan, and Bushehr provinces in Iran [5]. Climatic conditions are one of the most critical variables of the natural environment. Regression analysis of the climatic factors and henna yield showed that these variables described 93% of henna yield changes. All climatic characteristics of the cultivated areas except the sea level, growth period temperature, and annual temperature have a positive relationship with henna yield. About 87% of henna yield changes can be described by two factors, including relative humidity and rainfall during the growth period. These two parameters are more effective in henna yield in cultivated areas. Also, soil nitrogen and phosphorus elements have been reported to have a significant role in henna yield. Rudbar-e-Jonub, Shahdad, and Qaleh Ganj are in Kerman province in the southeast of Iran, located at latitude 30.29 and longitude 57.06 with an area of 180,726 km^2^. The average rainfall in Kerman is 138 mm. Rainfall deficiency and high evaporation rate make Kerman a dry area. Kerman’s climate is different due to high evaporations and special climatic conditions in different regions. These different climatic conditions can affect the secondary metabolites of plants [5].

*L. inermis* contains carbohydrates, flavonoids, phenolic, proteins, saponins, terpenoids, alkaloids, xanthones, resin, quinones, coumarins, fat, and tannins. Henna contains 2-hydroxy-1, 4-naphthoquinone (Lawson), and gallic acid [6]*.* Pharmacological studies have shown that *L. inermis* has antibacterial, antifungal, antiparasitic, analgesic, anti-inflammatory, wound and burn healing, anticancer, and many other pharmacological effects [7]. The whole plant, roots, leaves, fruits, stem, rhizome, barks, inflorescence, latex, bulbs, seeds, flowers, and oil are applied for the treatment of various diseases [8]. In Persian medicine (Iranian traditional medicine) sources, henna oil is recommended for infectious diseases, such as cervical ulcers [9]. *Streptococcus agalactia* and *Pseudomonas aeruginosa* cause cervicitis [10, 11].

*S. agalactiae,* also called *B Streptococcus* (GBS), is a Gram-positive coccus [12]. GBS as a harmless commensal bacterium is part of the human microbiota that is colonized in the genitourinary and gastrointestinal tract of 30% of healthy adults (carriers with no symptoms). However, GBS causes severe invasive infections, particularly in the elderly, infants, and those with compromised immune systems [13]. *S. agalactiae* is a major neonatal pathogen [14]. Group B *streptococcus* causes neonatal sepsis as well as meningitis in most countries [15].

*P. aeruginosa,* as a common facultatively aerobic Gram-negative bacterium, causes disease in animals, plants, and humans [16], which has become a health concern, in particular, in immunocompromised and critically ill patients. The drug-resistant strains cause high mortality [17]. It is the most common colonizer of medical devices, such as catheters. *P. aeruginosa* is transmitted by contaminated devices that are not appropriately cleaned or those in the hands of healthcare staff [18].

*Trichomonas vaginalis,* as a flagellated protozoan parasite in the human genital tract, is the cause of remediable sexually transmitted diseases worldwide. Genital tract infections in females can cause several symptoms, such as cervicitis and vaginitis. Recently, *T. vaginalis* infection is associated with several serious conditions, like cervical cancer, prostate cancer, adverse pregnancy outcomes, and a high likelihood of HIV infection; attempts have been made to treat and diagnose patients harboring *T. vaginalis* [19].

So far, no detailed study has been done on the effect of anti-microbial and anti-parasite activity of henna in various landraces. We investigated the phytochemical attributes and antimicrobial impact of the extracts from Shahdad, Rudbar-e-Jonub, and Qhaleh Ganj landraces, as local henna landraces.

## Methods

### Chemicals

Gallic acid, Lawson (2-hydroxy 1,4 naphthoquinon), Folin-Ciocalteu reagent, sodium carbonate, NaCl, blood agar, and Muller Hinton agar were purchased from Merck company. Ciprofloxacin was purchased from the Farabi company.

### The plants’ collection and extraction process

The local landraces of *L. inermis,* including Shahdad, Rudbar-e-Jonub, and Qhaleh Ganj were gathered and authenticated by the Medicinal and Industrial Research Institute, Ardakan. The voucher numbers are SSU 0062, SSU 0061, and SSU 0066, respectively. The dried leaves were sieved to prepare henna powder.

We prepared the hydroalcoholic extract through the maceration method. *L. inermis* leaves were ground into a fine powder, passed through the sieve, and macerated separately at 10 g of ground plant material in 70% (v/v) ethanol (80 ml) for 72 h. Extraction was performed at room temperature while shaking using a magnetic stirrer. The solution was then purified using the Buchner funnel and concentrated. The concentrated extract was stored far from the light and heat [20].

### Total phenol content

The Folin–Ciocalteu method was used to determine the total phenolic content of extracts and oil. Gallic acid was applied as a standard, and total phenol was represented as mg/g of gallic acid equivalents (GAE). GA at 10, 20, 40, 60, 80, 100, and 200 μg/ml concentrations was prepared, followed by mixing with 0.5 ml of a 10-fold diluted Folin-Ciocalteu reagent as well as 0.4 ml of 7.5% sodium carbonate after 3–8 min. Parafilm was used to cover the tubes and they were kept for 30 min at room temperature before reading the absorbance at 760 nm spectrophotometrically. All assessments were done in triplicate. Total phenolic content was calculated as mg of GA per gram using the equation achieved from a standard GA calibration curve [21].

### Preparation of the bacterial strains

The commercial strains of *P. aeruginosa* ATCC 27853 and *Streptococcus* B PTCC1864 were prepared using the Laboratory of Industrial Microbiology, Shahid Sadoughi University of Medical Sciences, Yazd, Iran.

Stock bacterium cultures were kept for 2 hours at room temperature. All strains were streaked on nutrient and blood agar plates, followed by incubation at 37 °C for 24 hours. The inoculums were provided by emulsifying at least three colonies from the plates in sterile 0.9% NaCl (w/v) until forming 10^8^ 10^8^ CFU/ml (0.5 McFarland scale). The sterile conditions of the procedures were assured using laminar hood equipment.

### Preparation of *trichomonas vaginalis*

The vaginal discharge of females with *Trichomonas vaginitis* referring to the healthcare centers of Yazd, Iran were used to isolate *T. vaginalis* strains, and were transferred to the TYI-S-33 culture medium, and stored in the University’s Parasitology Research Laboratory until usage. *T. vaginalis* cells were collected from the logarithmic growth phase and the number of cells was estimated using a hemocytometer slide. Then, 1 × 10^5^ 10^5^ /ml cells were applied for the anti-*T. vaginalis* impacts of *L. inermis* landraces.

### Antibacterial effects

The standard bacteria (*P. aeruginosa* and *S. agalactiae*) were passaged on a blood agar medium and incubated at 37 °C for 24 hours. For the agar well diffusion method, the Muller Hinton agar plate was covered with bacterial suspension, and wells were created with a sterile Pasteur pipette in each plate with a 6-mm diameter. Then, 50 μL of various concentrations of plant extracts were transferred to the respective wells in the plate media. Ciprofloxacin was employed as a positive control and sterile distilled was applied as a negative control. The zones of inhibition were measured by a ruler and recorded (in mm). All tests were performed in triplicate.

### Determination of minimal inhibitory concentration (MIC)

MIC assessments were done using the broth micro-dilution technique and were performed in Mueller Hinton broth (MH), according to the National Committee for Clinical Laboratory Standards (NCCLS 1999b) [22]. After preparing serial dilutions of plant extracts (8, 10, 20, 30, and 40 mg/ml) in MH broth, each dilution (50 μL) was dispensed into the wells, followed by inoculation with 25 μL of the bacterial suspension (0.5 Macfarland) and 25 μl MH broth and mixing completely. Negative (growth) and positive (sterility) controls were applied for all experiments. Bacterial growth was controlled by replacing the extract with ethanol 10% (the same volume) for eliminating the probable antibacterial activity of the solvents. MH broth medium was used for preparing sterility controls. The ultimate volume in wells was 100 μl. After covering the plates with a sterile plate sealer, they were subjected to incubation at 37 °C for 24 hours. Following incubation, MIC was regarded as the lowest sample level with no color change (clear) and showing complete inhibition of bacterial growth.

### Minimal bactericidal concentration (MBC)

For determination of the MBC, 10 μl of broth aliquots was obtained from each well with an extract level higher than or equal to the MIC values, followed by incubation in MH agar at 37 °C for 24 hours. Each experiment was done three times.

### In vitro anti-Trichomonal assay

For evaluating the anti-Trichomonal impacts of Henna extracts, 0.013–26.6 μg/ml of the extract concentrations were disposed in phosphate buffer saline (PBS) and mixed in the microtubes. PBS and metronidazole (50 μg/ml) were applied as negative and positive controls, respectively. Afterward, 100 μl of medium with about 10^5^ live *T. vaginalis* microorganisms were transferred to each tube and they were subjected to incubation at 37 °C, and the count of live parasites in each tube was calculated 24, 48, and 72 hours after incubation. For all samples and each time, after shaking the tube, the live cells were calculated by a hemocytometer slide. The active parasites, as well as parasites with moving flagellum, were regarded as alive. All experiments were done in triplicate. The count of live parasites was compared with the negative and positive controls. After calculating the growth inhibitory percentage (GI %), it was reported using the following formula:

$$GI\%=a-b/a(100)$$

Where, a is the mean of live parasites in the negative control tube and b indicates the mean of live parasites in the test tube [23].

### Statistical analysis

The results were computerized and analyzed by SPSS 25. One-way ANOVA and Tukey’s test were used to analyze the results.

## Results

### Total phenol

Total phenolic content of henna landraces were calculated by the standard curve of gallic acid. Table 1 reveals that the total phenol content of Shahdad, Rudbar-e-Jonub, and Qaleh Ganj was 206.51 ± 0.07, 201.96 ± 0.09, and 254.85 ± 0.01 μg/ml, respectively and their difference was significant (*P* < 0.05).

**Table 1:** Zone of growth inhibition of Henna landraces against GBS and *P. Auroginosa* and content of Phenols

Landraces Conc.(mg/ml)	ZGI (cm) of GBS			ZGI (cm) of ***Psodumonas auroginosa***		
	Shahdad	Roodbar-e-Jonoob	Ghale-e-ganj	Shahdad	Roodbar-e-Jonoob	Ghale-e-ganj
**3**	–	–	0	–	–	–
**4**	0	0	–	0	0	–
**6**	–	–	1.06 ± 0.1	–	–	–
**8**	0	0	1.23 ± 0.05	1.43 ± 0.05	0.96 ± 0.05	0.86 ± 0.11
**10**	0.76 ± 0.05	0	1.33 ± 0.15	1.56 ± 0.15	1.13 ± 0.11	1.16 ± 0.15
**15**	–	–	1.5 ± 0.1	–	–	–
**20**	0.9 ± 0.1	0.7 ± 0	1.7 ± 0.1	1.7 ± 0.1	1.4 ± 0.2	1.46 ± 0.05
**30**	1.13 ± 0.05	0.8 ± 0	–	1.96 ± 0.05	1.73 ± 0.05	–
**40**	1.26 ± 0.05	0.97 ± 0.05	2.06 ± 0.05	2.46 ± 0.15	1.93 ± 0.2	1.66 ± 0.05
**Phenol content** (μg/ml)	206.51 ± 0.07	201.96 ± 0.09	254.85 ± 0.01	–		

*ZGI* Zone of growth inhibition, *GBS* group B Streptococcus

### Antimicrobial effect

The growth inhibition of henna landraces against GBS and *P. auroginosa* is shown in Table 1. There was no growth inhibition at the concentration of 4 mg/ml of Shahdad and Rudbar-e-Jonub samples and no growth inhibition at the concentration of 3 mg/ml of Ghaleh Ganj samples.

**Fig. 1:**
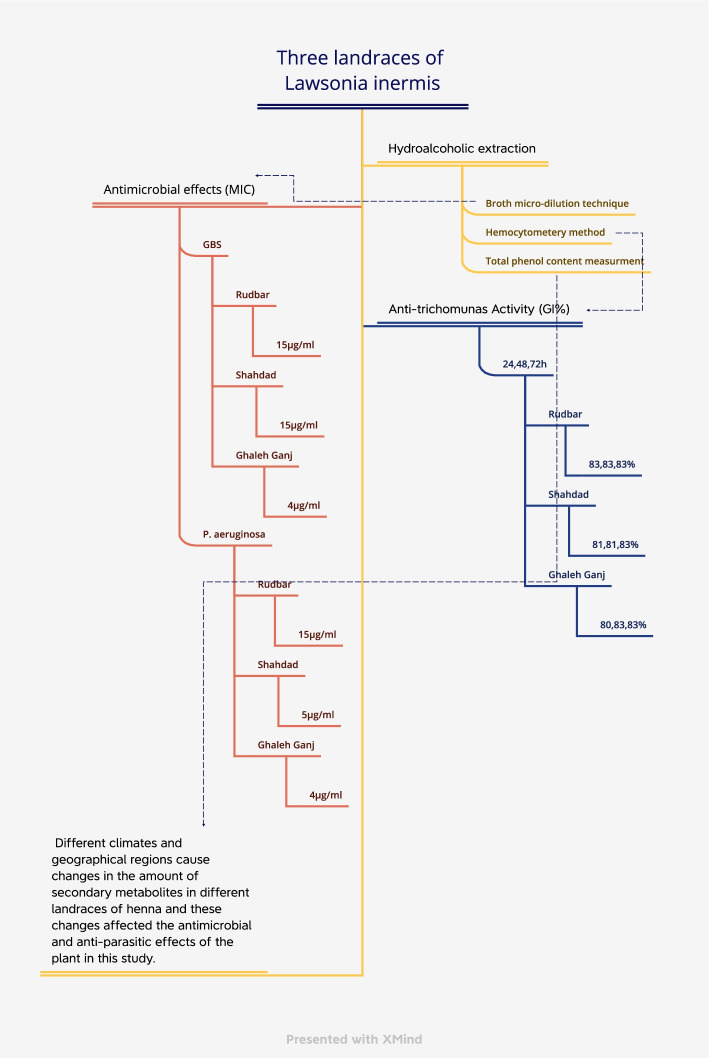
The study at a glance (Growth inhibitory percent (GI%), MIC: Minimum Inhibitory Concentration (μg/ml), GBS: Group B streptococcus)

None of the henna landraces showed MBC, but those from Shahdad, Rudbar-e-Jonub, and Qaleh Ganj had MIC against GBS at 15, 15, and 4 μg/ml. Also, they had MIC against *P. auroginosa* at 5, 15, and 4 μg/ml (Table 2) (Fig. 1). Positive control was ciprofloxacin at the concentration of 12.5 mg/ml. Negative control was medium and bacteria.

**Table 2:** Minimum Inhibitory Concentration (μg/ml) of Henna landraces against GBS and *P. auroginosa*

Landraces	Shahdad		Roodbar		Ghale ganj	
Bacteria Cells	*GBS*	*P.auroginosa*	*GBS*	*P.auroginosa*	*GBS*	*P.auroginosa*
**A**	4	4	4	4	3	3
**B**	5	5	5	5	4	4
**C**	10	10	10	10	5	5
**D**	15	15	15	15	7.5	7.5
**E**	20	20	20	20	10	10
**F**	C(−)		C(−)		C(−)	
**G**	C(+)		C(+)		C(+)	
**H**	Medium		Medium		Medium	

*Conc.* concentration, *C(+)* positive control: ciprofloxacine, *C(−)* negative control: bacteria and medium, *GBS Group B* Streptococcus the blacked cell showed bacteria growth

### Anti-*T. vaginalis* effect

The maximum GI% of Shahdad extract was 81 ± 0.36%, 83 ± 0.4%, and 83 ± 0.4% after 24, 48, and 72 h, while the maximum GI% of the Rudbar-e-Jonub extract was 83 ± 0.6%, 83 ± 0.3%, and 83 ± 0.4% and that of Qaleh Ganj extract was 80 ± 1%, 83 ± 0.3%, and 83 ± 0.4%, respectively.

GI% of MTZ was 100%. The difference between GI% at 24, 48, and 72 h in three landraces was not significant (*P* > 0.05). At 48 and 72 h, the difference between all concentrations with MTZ was significant (*P* < 0.05). The maximum GI% in three landraces at 24, 48, and 72 h was different significantly and the GI of Rudbar-e-Jonub extract (83%) was higher than others (*P* < 0.05) (Fig. 1).

**Fig. 2:**
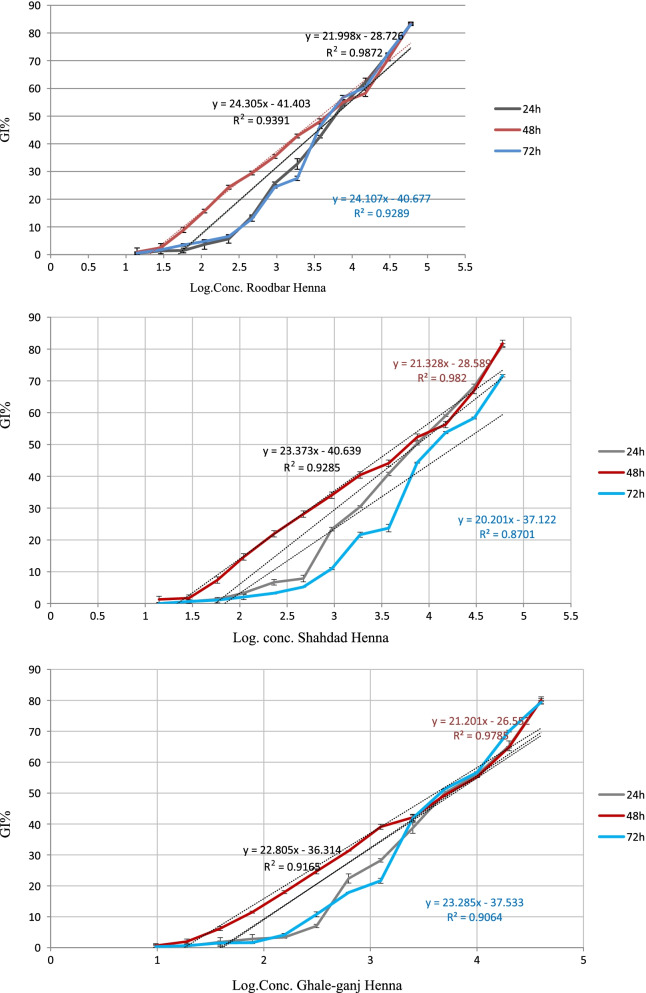
Growth inhibitory percent (GI%) of *T. vaginalis* vs. logarithm (Log) of concentration of extracts of Henna landraces. GI%: percentage of growth inhibitory of parasit

As shown in Fig. 2, IC50 of Shahdad Henna extract after 24, 48, and 72 h was 7.54, 4.83, and 20.54 μg/ml, respectively. IC50 of Rudbar-e-Jonub extract was 5.76, 3.79, and 5.77 μg/ml, and IC50 of Qaleh Ganj extract was 6.09, 4.08, and 5.74 μg/ml on different days (Fig. 2).

The maximum GI% of Shahdad extract was 81 ± 0.36%, 83 ± 0.4%, and 83 ± 0.4% after 24, 48, and 72 h, while the maximum GI% of the Rudbar-e-Jonub extract was 83 ± 0.6%, 83 ± 0.3%, and 83 ± 0.4% and that of Qaleh Ganj extract was 80 ± 1%, 83 ± 0.3%, and 83 ± 0.4%, respectively.

**Fig. 3:**
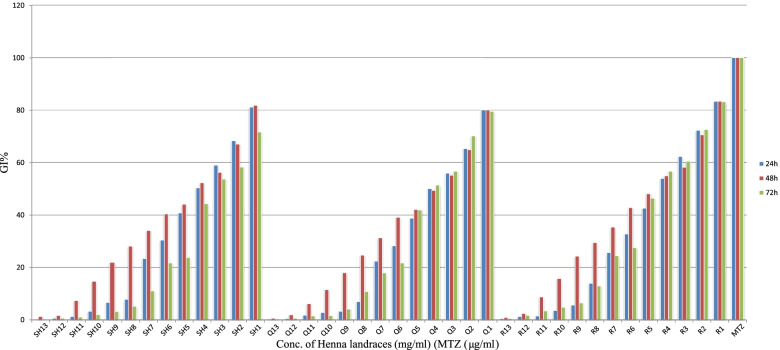
The inhibitory effects of Henna landraces extract, Shahdad (SH1-SH13: 0.013–26.6 μg/ml), Qaleh Ganj (GH1-GH13: 0.013–26.6 μg/ml), Rudbar-e-Jonub (R1-R13: 0.013–26.6 μg/ml), and Metronidazole (50 μg/ml) on *T. vaginalis* growth in different concentrations and incubation times. GI%: percentage of growth inhibitory of parasit

The results of comparing the GI% of *T. vaginalis* can be seen in Fig. 3.

## Discussions

We found that different landraces of *L. inermis* have various effects against GBS and *P. auroginosa*. The MIC of Shahdad, Rudbar, and Qaleh Ganj against *P. auroginosa* was 5, 15, and 7.5 μg/ml, respectively (Table 2). Rudbar-e-Jonub landrace showed an antimicrobial effect against GBS at 4 μg/ml but it had a smaller GI diameter than other landraces. However, Qaleh Ganj landrace at 4 μg/ml showed an antimicrobial effect against GBS but it had a bigger GI diameter than other landraces. Also, the Qaleh Ganj landrace had a bigger GI against GBS than *P. auroginosa* (Tables 1 and 2). *P. aeruginosa* is a non-fastidious microorganism with no need for special cultivation conditions. This bacterium is a common cause of infection among non-fermenting Gram-negative bacteria, mainly influencing immunocompromised patients. Increased prevalence of resistance leads to prolonged therapy and high mortality [24]. Habbal et al. reported that different landraces of henna had higher antimicrobial effects against *P. auroginosa* than other microorganisms [25].

Qaleh Ganj had higher and Rudbar-e-Jonub had lower total phenol content between the three landraces. Phenolic content in henna are ferulic acid, gallic acid, and luteolin [26]. Luteolin (3,4,5,7-tetrahydroxyflavone) as a flavon is highly available as a secondary metabolite in plants. It has many pharmacological properties, like anti-inflammatory, anticancer, anti-allergic, antioxidant, and antimicrobial. It presents favorable antibacterial activity against *Staphylococcus aureus, Bacillus subtilis, Escherichia coli*, *Listeria monocytogenes,* and *Pseudomonas fluorescens* [27]*.*

In this study, Shahdad and Qaleh Ganj landraces had a greater antibacterial effect against Gram-negative bacteria than Gram-positive bacteria. This effect may be related to Lawson. Lawson (2-hydroxy 1,4 naphthoquinone) is a naphthoquinone that is found in henna. Gram-negative bacteria are more resistant to antimicrobial agents compared to Gram-positive bacteria due to their cell walls. Gram-positive bacteria have a porous and thick cell wall with inter-connected peptidoglycan layers surrounding a cytoplasmic membrane, whereas Gram-negative ones possess a thinner peptidoglycan layer, an outer membrane, and a cytoplasmic membrane. Gram-positive bacteria have a porous layer of peptidoglycan as well as a single lipid bilayer, whereas Gram-negative ones possess a double lipid bilayer that sandwich the peptidoglycan layer and also an outer layer of lipopolysaccharide, leading to a low level of permeability for lipophilic small molecules [28].

Hydroxyl (−OH) group of phenolic compounds possibly causes bacterial inhibition and the double bonds (position and number) can cause an antimicrobial effect. Two carbonyl groups in an aromatic ring as part of the naphthoquinone structure could describe antimicrobial effects. This hypothesis is supported by the oxygen reduction activity of the quinone structure in 2-hydroxy-1,4-naphthoquinone with the production of reactive oxygen species (ROS) and damage of macromolecules, like proteins, DNA, and lipids [29]. Chemical compounds target the infection-inducing bacterial cells; ROS production is an important process in apoptosis. The mechanism of such antibacterial agents is elevated ROS production and consequently, apoptotic cell death. Regarding the ability to produce ROS, naphthoquinone analogues are very cytotoxic for the infected cells and are capable of restricting cellular enzymes involved in cell growth and apoptosis [30].

Paiva et al. in 2003 reported that Plambajine, a naphthoquinon, had an antimicrobial effect against *Psudomunas*, *Basilus sabtilis*, *Proteus vulgaris*, and *Candida* [31]. Karkare et al. in 2013 reported that Diosperin, a naphthoquinone, inhibited *Mycobacterium tuberclusis* by DNA gyrase inhibition [32].

Under biotic/abiotic stress conditions, the composition of plants and their extracts change and cause them to have different effects on the same microorganisms [33]. Because of climate diversity in Kerman province, total phenol and Lawson content of different varieties of henna are different; thus, they have different effects on microorganisms. In this study, Qaleh Ganj extract had a greater GI diameter against GBS, and Shahdad extract showed a greater GI diameter against *P. auroginosa*.

According to the results of anti-*Trichomunas* activity, all evaluated extracts showed effectiveness in preventing the growth of *T. vaginalis* trophozoites dose-dependently after 24, 48, and 72 h of incubation. Moreover, the Rudbar-e-Jonub henna significantly showed more effectiveness due to lower IC50 values for trophozoites of *T. vaginalis* after 24 h (*P* < 0.05) (Fig. 2).

The maximum GI% of the Shahdad, Rudbar-e-Jonub, and Qaleh Ganj landraces was 81, 83, and 80% after 48 h, respectively (Fig. 2). The GI% of Rudbar-e-Jonub extract was significantly different compared to two other landraces after 24, 48, and 72 h; but the total phenol in Rudbar was lower than others significantly (*P* < 0.05). Therefore, other constituents of henna have a role in its anti-Trichomunas effect.

Multidrug resistance is one of the causes of human mortality in the past few years. Bacteria, parasites, and fungi have numerous resistant mechanisms against the current antibiotics, causing severe effects on patients’ health. In addition, the use of synthetic chemicals to control microorganisms is still limited due to their environmental and carcinogenic effects and acute toxicity. Hence, a demand for new antibiotics is urgently raised in the scientific community to deal with multidrug resistance. The therapeutic agents from herbs have long emerged as a potential natural source for treating infectious diseases [34, 35]. Some studies have reported anti-*Trichomunas *effect of essential oils such as *Atalantia sessiflora* [35, 36]. Motazedian et al. and Serakta et al. declared the anti-leishmania effect of henna extract [37, 38]. The ethyl extract (33 mg/L) and petroleum ether extract (27 mg/L) of henna have an anti-falciparum effect [39].

In this study, the extract was not dissolved further, otherwise, it might have shown a higher GI. The IC50 of Shahdad extract after 72 h was more than others, More studies are needed for the detection of constituents responsible for this difference.

## Limitations

The most important limitation of this study was the impossibility of preparing a more concentrated extract solution.

## Conclusions

Different climates and geographical regions cause changes in the amount of secondary metabolites in different landraces of henna and these changes affected the antimicrobial and anti-parasitic effects of the plant in this study.

## Data Availability

The datasets used and/or analyzed during the current study are available from the corresponding author on reasonable request.

## Abbreviations

GBS: *B Streptococcus*
GA: Gallic acid
MH: Mueller Hinton broth
PBS: Phosphate buffer saline
GI %: Growth inhibitory percentage
